# The use of technology in creating individualized, meaningful activities for people living with dementia: A systematic review

**DOI:** 10.1177/1471301220928168

**Published:** 2020-05-31

**Authors:** Gemma Goodall, Kristin Taraldsen, J Artur Serrano

**Affiliations:** Norwegian University of Science and Technology, Trondheim, Norway; Norwegian University of Science and Technology, Trondheim, Norway; Norwegian Centre for eHealth Research, University Hospital of North Norway, Tromsø, Norway

**Keywords:** dementia, technology, psychosocial, person-centered, systematic review

## Abstract

There is a growing interest in using technology to provide meaningful activities for people living with dementia. The aim of this systematic review was to identify and explore the different types of digital technologies used in creating individualized, meaningful activities for people living with dementia. From 1414 articles identified from searches in four databases, 29 articles were included in the review. The inclusion criteria were the study used digital technology to deliver an individually tailored activity to participants with dementia, the process of individualization was described, and findings relating to the mental, physical, social, and/or emotional well-being of the participant were reported. Data extracted from the included studies included participant demographics, aims, methods, and outcomes. The following information on the technology was also extracted: purpose, type, training, facilitation, and the individualization process. A narrative synthesis of the results grouped the various technologies into four main purposes: reminiscence/memory support, behavior management, stimulating engagement, and conversation/communication support. A broad range of technologies were studied, with varying methods of evaluation implemented to assess their effect. Overall, the use of technology in creating individualized, meaningful activities seems to be promising in terms of improving behavior and promoting relationships with others. Furthermore, most studies in this review involved the person with dementia in the individualization process of the technology, indicating that research in this area is adopting a more co-creative and inclusive approach. However, sample sizes of the included studies were small, and there was a lack of standardized outcome measures. Future studies should aim to build a more concrete evidence base by improving the methodological quality of research in this area. Findings from the review indicate that there is also a need for more evidence concerning the feasibility of implementing these technologies into care environments.

## Introduction

Dementia is an umbrella term for various neurodegenerative syndromes that impact primarily memory, cognition, language, and behavior. There are currently around 50 million people living with dementia worldwide, and it is estimated that there are almost 10 million new cases of dementia each year (Prince et al., 2015). Given the increasing prevalence and incidence of dementia, the World Health Organization (WHO) has stressed the need to invest in research and cost-effective approaches to meet the needs of people living with dementia and their caregivers (WHO, 2015).

Psychosocial approaches to supporting those living with dementia include the use of meaningful activities to promote well-being. Previous literature that aims to define the term “meaningful activity” in the context of dementia care has often done so from the perspective of people with dementia, their family, and health-care professionals (Harmer & Orrell, 2008; Phinney et al., 2007). Focus is placed on values and beliefs that resonate with past roles, interests, and routines of the individual with dementia. Harmer and Orrell (2008) categorized activities considered to be “meaningful” into reminiscence, family and social, musical, and individual activities. This literature review focuses on the last of these activities, although the four types tend to overlap. Harmer and Orrell (2008) describe individual activities as being adapted to the preferences and capabilities of the person with dementia, and discuss the importance of relating these activities to the past lifestyle of the individual. This review uses the term “individualized” to emphasize that the fact that a process has taken place to adjust the activity to the specific preferences and abilities of the individual.

While work in this field has long focused on person-centered care (Brooker, 2003; Kitwood, 1997), findings from previous literature reviews concerning the individualization of activities for people with dementia appear to be mixed. Travers et al. (2016) recommend that individualized activities may be effective for behavioral and psychological symptoms of dementia, especially with regard to improving passivity and agitation, and increasing pleasure and interest. Subramaniam and Woods (2012) conducted a systematic literature review on the impact of individual reminiscence therapy for people with dementia. The authors suggest that conducting a life review with a person with dementia, in which a life storybook is produced, has a positive impact on cognition and well-being. They also suggest that personhood and well-being can be promoted using individualized reminiscence approaches that meet specific needs of the individual with dementia. Despite these suggestions, however, a recent Cochrane review found very little evidence for personally tailored activities being able to improve psychosocial outcomes for people living with dementia (Möhler et al., 2018). While offering personally tailored activities (such as listening to individualized music playlists or making puzzles from familiar photographs) to people with dementia in long-term care may slightly improve challenging behavior, effects on mood were uncertain, and the authors were unable to make recommendations about specific activities.

Constant advances in technology provide potential for designing new and innovative ways of meeting specific needs of individuals with dementia. In a very recent overview of technology and dementia, Astell et al. (2019) identified leisure and activity as one of the main domains of technology development within dementia care. The authors remark that technology—such as smartphones, tablets, wearables, robots, virtual reality, and artificial intelligence—is prompting thought on how care services can be better delivered to address the well-being of people with dementia. The authors also argue that the rapid pace of technology development requires a holistic view of dementia. In expanding the view of dementia beyond a narrow medical approach, technology may be used to empower people with dementia, supporting them to live a more meaningful life. For instance, a recent study suggested that the use of a social robot for hospital patients with dementia promoted a sense of self and facilitated social connection with others (Hung et al., 2019).

Arthur (2009) defines technologies as assemblies of practices and components put to use in order to fulfill a specific purpose. In recent years, there has been much work done on the use of various technologies for providing meaningful activities in dementia care. Digital technologies, such as mobile and tablet apps, have been suggested to enable collaborative explorations of life events by people with dementia and caregivers, encouraging the caregiver to reflect and learn more about the individual (Maiden et al., 2013). Purves et al. (2011) also comment on the role that multimedia technologies (e.g., digital life stories) have on conveying the narrative of people living with dementia, and the authors stress that further work needs to be done in understanding how these technologies can be used in everyday practice. In a review on touchscreen technology for people with dementia, Joddrell and Astell (2016) commented that the primary use of touchscreen technology has been to deliver assessments and screening tests, and they called for more focus on how this technology can be used to deliver independent activities for meaningful occupation.

To date, there are no literature reviews that provide an overview of the evidence on using technology to create individualized, meaningful activities for people with dementia. Furthermore, there is arguably a need to take qualitative and mixed-method studies into account in this area of research, especially given that meaningful activity within dementia care is often measured in subjective terms of enjoyment (Harmer & Orrell, 2008). While the importance of thorough quantitative meta-analyses remains, much can be learnt from qualitative and mixed-method research in addition to quantitative studies. The Cochrane Qualitative and Implementation Methods Group Guidance Series highlights the important role of qualitative and mixed-method reviews in understanding how interventions work and how they are implemented (Noyes et al., 2018). Therefore, this literature review will consider qualitative, quantitative, and mixed-methods research to acquire a comprehensive overview of the work that has been done on this topic.

The main purpose of this review is to answer the following research question: What are the different digital technologies used to create individualized activities for people with dementia, and how are these facilitated? For the purpose of this review, we define digital technologies as devices, systems, or applications that can be used to create, store, view and/or share information electronically. In order to further explore the findings from this question, the review will also answer the following secondary research questions: (a) How are these technologies individualized? and (b) What is known about the effects of these technologies on the well-being of people living with dementia?

## Methods

This systematic review was conducted and reported in accordance with the Preferred Reporting Items for Systematic Reviews and Meta-Analyses (PRISMA) guidelines (Moher et al., 2009a).

### Eligibility criteria

The SPIDER strategy (Sample, Phenomenon of Interest, Design, Evaluation, Research type) was used as a tool for shaping the search. SPIDER has been adapted from the PICO formulation (Population, Intervention, Comparison, Outcome) to be more suitable for qualitative and mixed methods research (Cooke et al., 2012). The SPIDER strategy for this review was as follows:

- Sample: people living with dementia.
- Phenomenon of interest: technology-based, meaningful activities tailored specifically for the person with dementia.
- Design: case study, observational study, randomized controlled trial, quasi-experimental study, questionnaire, interviews, and focus groups.
- Evaluation: outcomes related to the mental, physical, social and/or emotional well-being of the person with dementia.
- Research type: quantitative, qualitative, or mixed-method.

Only studies published in a peer reviewed journal and in English language were considered for review. In order to focus on more recent technologies, studies published before 2005 were not considered for review. Additionally, as another systematic review focusing on meaningful interventions for people living with dementia noted, person-centered care practices were not widely adopted until 2005 (Travers et al., 2016). Given that the scope of this review is to focus on individualized activities, it was deemed appropriate to limit the results to being published in 2005 or later.

#### Inclusion criteria

Studies were included if they met all of the following criteria: (a) uses digital technology to deliver an individually tailored activity to participants with dementia, (b) describes the process of individualization, and (c) reports on findings directly relating to the mental, physical, social, and/or emotional well-being of the person with dementia.

#### Exclusion criteria

Studies were excluded if they met any of the following criteria: (a) reports solely on the well-being of caregivers or (b) reports findings solely relating to the technology rather than the person with dementia. Literature reviews, study protocols, theoretical papers, conceptual papers, and position papers were also excluded from the review.

### Information sources

Given the interdisciplinary nature of this topic, four databases were used for the search, with the aim of capturing as many potential articles as possible. The following databases were used: CINAHL, Embase, PubMed, and Scopus. A combination of Boolean operators and truncations were used. MeSH Terms were also used where applicable. Table 1 gives a summary of the search terms.

**Table 1.** Summary of search terms.

Search	Terms
#1	dement* OR alzheimer’s
#2	personal OR personalized OR personalised OR person-centred OR person-centered OR person-focused OR individualized OR individualised OR individualistic OR meaningful OR biographical OR autobiographical OR tailored
#3	technology OR virtual OR augmented OR media OR multimedia OR touchscreen OR iPad OR app OR mobile OR ICT OR tablet*
#4	#1 AND #2 AND #3

### Search

#### Study selection

The selection of articles for review was conducted by the first author. All articles underwent a first screening after duplicates were removed. This consisted of titles and abstracts being screened against the inclusion and exclusion criteria. Included articles then underwent an assessment for eligibility, which involved a reading of the article in full. Additionally, backward citation searching and forward citation tracking was conducted on these articles. Articles from this additional search that met the inclusion criteria were included for review. Coauthors Kristin Taraldsen and J Artur Serrano independently checked the final selection of articles against the inclusion and exclusion criteria.There were no discrepancies, and therefore this final selection of articles was approved by all authors.

#### Data extraction and synthesis

Data relating to the study aims, design, demographics, data collection, methods, and findings were extracted from each article. Additionally, information on the purpose of technology studied, type of technology, media contents and services, the individualization process, environment of technology use, training on technology use, and facilitation of the intervention/activity was also extracted.

Due to the heterogeneity of the results and the novelty of this field of research, no meta-analysis was conducted. The application of technology for meaningful activities is still an emerging area of work, with many different approaches and devices being used. Therefore, results are presented through a narrative synthesis. Findings from the studies are summarized to answer each of the research questions in turn.

## Results

### Study selection

**Figure 1.**
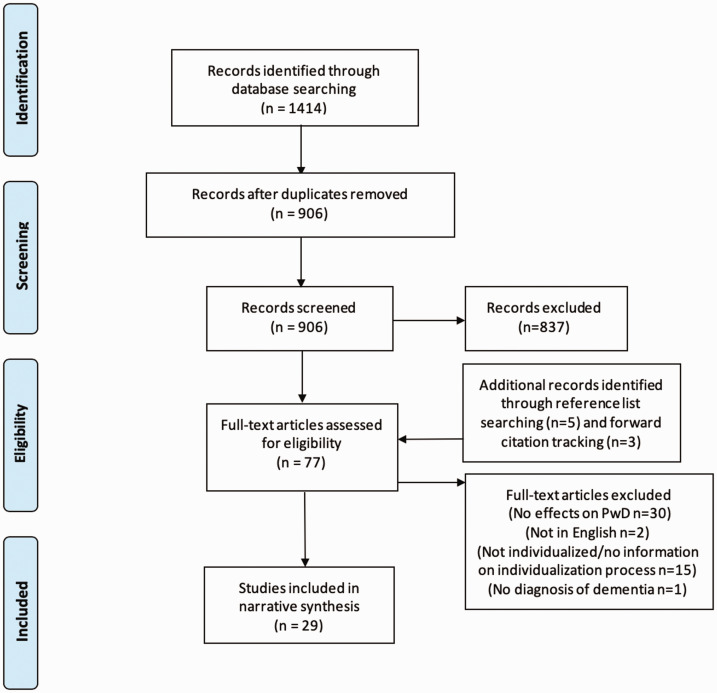
PRISMA flow diagram of study selection process. Adapted with permission from Moher et al.(2009b).

The initial search returned 1414 articles: 217 from PubMed, 507 from Scopus, 139 from CINAHL, and 551 from Embase. An overview of the study selection is shown in Figure 1. In short, 906 records were screened and 837 were excluded. Reference list checking and forward citation tracking was conducted on the remaining 69 articles to identify additional records. From these searches, 8 articles were identified, meaning that a total of 77 articles were assessed for eligibility. This assessment resulted in a total of 29 articles for review.

### Study characteristics

Twenty-nine studies (reported in 29 separate articles) were included for review. From these studies, 12 were qualitative, 13 used mixed-methods, and 4 were quantitative. An overview of study characteristics is given in Table 2, which summarizes study design, participant information, aims of the study, interaction with technology, measures, and findings for each study.

**Table 2.** Study characteristics.

Author	Study design	Participants with dementia			Study aim(s)	Interaction with technology	Measures	Findings
		N	Mean age	Type and severitya				
Critten and Kucirkova (2017)	Case study	3	83.3	Mild to moderate dementia	Study the role of an individualized multimedia iPad app (Our Story) in the stimulation, preservation and sharing of special memories	Creation of multimedia story over 7 weeks	Interviews, field notes, observations	The app facilitated the elicitation, storage and sharing of special memories. All participants experienced positive feelings of confidence, empowerment and increased self-esteem
Damianakis et al. (2010)	Exploratory feasibility study	12	79.6	Early to advanced AD (N = 6), MCI (N = 6)	Observe participant responses to personalized multimedia biographies	Production of DVD biographies followed by weekly screenings for 6 months	Observations, interviews	Multimedia biographies stimulated reminiscence, evoked feelings of mostly joy but occasionally sadness, and stimulated social interactions
Davis and Shenk (2015)	Mixed-methods study	10	84.3	Late moderate dementia, mean FAST score 5.2	Compare effects of personalized and generic multimedia videos on engagement	Video screenings over 6 weeks	OME, language patterns from transcriptions using predefined codes	Slight preference for looking at personalized videos first. Generic videos produced wider range of conversational topics and phrasal patterns.
Davison et al. (2016)	Randomized, single-blinded cross-over study	11	86	Mild to severe dementia. Mean MMSE 16.2	Assess the effects of a personalized multimedia system (Memory Box) on agitation, depression and anxiety	Use of multimedia system for 4 weeks	CMAI; CSDD; RAID, interviews	Significant reduction in depression and anxiety scores. No significant change in agitation scores
De Leo et al. (2011)	Case study	1	80	AD, FAST Stage 4	Assess whether a slideshow of daily life moment pictures captured by a smart phone can support autobiographical memory	Use of smart phone for 4 weeks. DVD of recent events viewed once a week	Recent events memory recall test (non-standardized) and satisfaction questionnaire	DVD of daily life pictures helped the participant recall recent events significantly better
Ekström et al. (2015)	Case study	1	52	AD	Explore the effects of a personalized digital device application (GoTalk NOW) on communication	Use of device over 2 weeks	Video recordings, interview	Application use increases interactive and communicative actions
Hashim et al. (2015)	Case study	1	74	Mild AD	To test a personalized digital memory book (myBook) for usability, functionality, reminiscence, and cognition	Weekly sessions over 8 weeks	Observations, evaluation form (non-standardized)	The participant felt the tool enhanced reminiscence. Observations indicated improvement in social interaction and communication
Hung et al. (2018)	Mixed-methods feasibility and acceptability study	4	Range 69–80	AD/Vascular/Parkinson’s Dementia	Explore the feasibility and acceptability of using an iPad Simulated Presence Therapy intervention	iPad simulated presence therapy over 2 weeks	Video ethnography, observations, semi-structured interviews with hospital staff	Four themes: (a) positive responses, (b) person-centered care, (c) video content, and (d) technical skills.
Karlsson et al. (2014)	Explorative multiple case study	7	Range 72–81	Alzheimer’s disease	Explore the process of acceptance and integration of a digital photograph diary as a tool for remembrance of and conversations about daily life events	Use of digital photograph diary over 22 weeks	Interviews, observations, field notes, MMSE, PGCMS, Free recall memory index, recognition memory index	The digital photograph diary contributed to increased communication, promoted relationships, and was perceived as a stimulating joint activity
Karlsson et al. (2017)	Explorative study	7	77	Alzheimer’s disease, Mean MMSE 22	Explore how sense of self and identity are manifested in narrations about recent events enabled by a digital photograph diary	Use of digital photograph diary over 3 months	Audio recorded communication sessions, observations, field notes	Two themes from discourse analysis: manifestations of sense of self and sense of self in relation to others. Digital photography diary supported communication
Kerssens et al. (2015)	Qualitative study	7	77	Dementia (N = 4), MCI (N = 3)	Test the usability, feasibility and adoption of a touchscreen computer intervention (The Companion)	Use of touchscreen computer intervention over an average of 31 days	Goal attainment, observations, interview	The Companion facilitated meaningful and positive engagement
Khosla et al. (2014)	Field trial	4	–	–	Measure the impact of a personalized assistive robot on emotional engagement	Use of assistive robot over 6 months	Video recordings of interaction, activity data, emotional responses, quality of robot experience survey	The robot had positive impact on emotional well-being and provided sensory enrichment and social connectivity.
Khosla et al. (2016)	Longitudinal field trial	5	–	–	Evaluate the impact of a personalized assistive robot in the context of home-based dementia care	Use of assistive robot over 3–6 months	Observational videos, interactional data, quality of robot experience survey	Positive experience with the robot, improvement in sensory enrichment
Laird et al. (2018)	Quasi-experimental feasibility study	30	79	Early to moderate dementia	Measure the effect of technology-enabled reminiscence iPad app (InspireD) on mutuality, quality of carer and patient relationship and subjective well-being	Use of the iPad app over 12 weeks	Mutuality Scale, QCPR, WHO-5	Significant increases in mutuality, quality of carer and patient relationship, and subjective well-being
Massimi et al. (2008)	Pre/post-test single case study	1	84	AD, moderate	Assess whether a biographical ambient display (Biography Theatre) improves autobiographical memory and sense of identity	Use of ambient display over 1 month	GDS-30; GAS, AES-I; SIP-AD; TST; AMI; MMSE; IQCODE; custom interviews and questionnaires	Improvement in apathy and positive self-identity. No improvement in autobiographical memory, anxiety, depression and general cognition
McAllister et al. (2020)	Case study	3	82	Lewy body disease, AD, dementia non-specific, moderate to advanced	To explore the use of a personalized iPad app (Memory Keeper) with regard to barriers, facilitators, benefits and incorporation into care.	Use of app over 6-month trial period	Field notes, focus groups and individual interview	Five themes: experienced and expected benefits of Memory Keeper; engagement of the person with dementia and their response to memory keeper; recruitment, media collection, set-up training and handover; use of memory keeper in long term care setting
Navarro et al. (2015)	Case study	1	70	AD, MMSE 17	Study the effect of an ambient display system (AnswerBoard) and mobile app (AnswerPad) on behavioral symptoms	Use of system over 16 weeks	NPI-Q; AES, caregiver diary, interviews	Use of systems had a positive effect in reducing challenging behaviors
Navarro et al. (2016)	Field study	2	N1 = “Over 70”, N2 = 73	AD, MMSE scores 17 and 21	Study the effectiveness of assisted cognition system (AnswerBoard/AnswerPad) to support occupational therapy interventions	Use of system over 16 weeks	NPI-Q; AES	Use of systems had a positive effect in reducing challenging behaviors
O’Connor et al. (2011)	Single-system ABA withdraw design	1	83	Vascular dementia, severe PAS = 21	To evaluate the effect of video-simulated presence for decreasing resistance to care	Video simulated presence for 14 days	Observations, adapted Positive Response Schedule	Significant reduction in resistance to care with the use of video simulated presence
Park et al. (2017)	Multi-site case study	7	–	Early stage dementia	Assess how digital storytelling affects quality of life in terms of relationships and self-identity	Seven-session workshop over 6 weeks	Observational field notes, audio recorded workshop sessions and interviews	Overall enjoyment of sessions, ability to share stories, enhance relationships, increase in communication and interaction
Peeters et al. (2016)	Formative evaluation	5	–	Early to severe dementia	Evaluate the use of a personalized music platform (Music ePartner)	Single time use of music platform	Observations, interview	Observed positive effects on memory recall, mood, and social interaction
Piasek et al. (2012)	Case study	1	87	Early-stage dementia	Explore whether SenseCam images can encourage meaningful discussions about recent memories and help maintain sense of identity	Use of SenseCam for 7 weeks. Viewing of images twice a week	Interview, observations, field notes, psychometric tests (not specified)	No change in psychometric measures, confusions about source of images, did not stimulate discussions
Ryan et al. (2018)	Qualitative study	15	78	–	To explore the impact of a personalized reminiscence program facilitated through an iPad app (InspireD)	Use of iPad app over 12 weeks	Semi-structured interviews	Six themes: usability, revisiting the past; home use; impact on the person with dementia; gains and abilities; impact on relationships
Samuelsson and Ekström (2019)	Dyad case studies	3	–	–	To understand how digital communication support may be used in interaction with people with dementia	Single time use of digital communication app	Video recordings	CIRCA and CIRCUS support conversation topics, personal photographs in CIRCUS are more engaging
Silva et al. (2017)	Single-blind randomized trial	51	73.7	Mild Alzheimer’s disease	To assess the effects of wearable camera memory aid (SenseCam) on well-being compared to paper memory training program (Memo+) and written diary (control)	Use of SenseCam for 6 weeks. Images reviewed twice a week	GDS-30, IAFAI, WHOQOL-OLD	Significant reduction in depression with SenseCam and Memo+ compared to diary control condition
Subramaniam and Woods (2016)	Multiple case study	6	82.2	Mild to moderate dementia, CDR scores of 1 (N = 5) and 2 (N = 1)	To compare the use of a multimedia digital life storybook vs. conventional life storybook	Digital storybooks created over 7–10 weeks, DVD then given to participants for regular viewing	QOL-AD; AMI-E; GDS-12R; QCPR; Semi-structured interview	Five out of six participants showed improvement in quality of life and autobiographical memory after having the digital storybook for 4 weeks
Welsh et al. (2018)	Case study	11	–	Moderate to severe	To explore whether a personalized digital app (Ticket to Talk) can support communication between people with dementia and younger people	Use of digital app over 4 weeks across 6 visits	Interviews, workshop discussions	Three themes: promoting and managing reminiscence; starting and maintaining conversation; redistributing agency
Woodberry et al. (2014)	Within-subject longitudinal study	6	72	AD, mild to moderate, Addenbrooke’s Cognitive Examination mean 70.8	To compare the effects of using a wearable camera vs. a written diary to aid retrospective recall of significant personal events	Use of wearable camera for 3.5 months	Memory recall tests, interview	Recall significantly more details of events in the camera condition for five out of six participants compared to the written diary condition
Yasuda et al. (2009)	ABCA design	15	77.3	AD, Mean MMSE 14.3	To assess whether personalized photo video obtain more attention compared to generic TV shows	Single event of viewing photo videos	Video recorded responses measured in terms of concentration and distraction scores	80% of participants showed more attention to personalized reminiscence photo video than other TV shows. Higher concentration scores for moderate and severe dementia participants

Note: Symbol “–” indicates not specified. AD: Alzheimer’s Disease; MCI: Mild cognitive impairment; FAST: Functional Assessment Staging Tool; OME: Observational measure of engagement; CMAI: Cohen-Mansfield Agitation Inventory; CSDD: Cornell Scale for Depression in Dementia; RAID: Rating Anxiety in Dementia; MMSE: Mini-mental state examination; PGCMS: Philadelphia Geriatric Center Morale Scale; QCPR: Quality of the Caregiver Relationship questionnaire; WHO-5: World Health Organization Five Well-being Index; GDS-30: Geriatric Depression Scale-30; GAS: Goldberg Anxiety Scale; AES-I: Apathy Evaluation Scale-Informant; SIP-AD: Self-Image Profile-Adults; TST: Twenty Statements Test; AMI: Autobiographical Memory Interview; IQCODE; Informant Questionnaire of Cognitive Decline in the Elderly; NPI-Q; Neuropsychiatric Inventory Questionnaire; AES: Apathy Evaluation Scale; PAS: Psychogeriatric Assessment scales; IAFAI; Adults and Older Adults Functional Assessment Inventory; WHOQOL-OLD: World Health Organization Quality of Life-OLD module; CDR: Clinical Dementia Rating scale; QOL-AD: Quality of life Alzheimer’s disease scale; AMI-E: Autobiographical Memory Interview extended version; GDS-12R: Geriatric Depression Scale (Residential).

^a^Type and severity as reported in the study.

The most commonly used study design was the case study (N = 12). Only two randomized trials were included. Other designs included field trials and explorative studies. A total of 231 participants were included across the 29 studies (ranging from 1 to 51, with a median of five participants per study). The mean age of participants ranged from 52 to 87. However, two studies only reported the age range of participants, and seven studies did not specify age. The severity of dementia varied across the studies, with all stages being covered from mild to severe. Two studies included participants with mild cognitive impairment in addition to participants with more advanced dementia. The most common type of dementia was Alzheimer’s disease (N = 14). There were inconsistencies in reporting participant demographics, with four studies failing to report either type or severity of dementia.

Most studies aimed to assess the impact of the technology-based activity on memory, communication or engagement. Some studies adopted a more exploratory approach and aimed to report any effects that the technology may have had on the person with dementia. Interviews and observations were the most popular tools for data collection, with thematic analysis and discourse analysis being used to draw findings. In quantitative and mixed-methods studies, there was a large variety of standardized measures used that focused on numerous domains (for details see Supplementary Material, Table S1). The studies were also greatly varied in terms of length of technology use, ranging from single-time use to use of the technology for six months. Across the 29 studies, the average time spent using the technology was seven weeks.

### Synthesis of results

#### What are the different technologies used to create individualized, meaningful activities for people with dementia, and how are these facilitated?

A wide array of technology, with varying media contents and services, has been explored for creating individualized, meaningful activities for people with dementia. This review categorized the technologies into four main purposes that all tackle common challenges people living with dementia face, namely: reminiscence/memory support, behavior management, stimulating engagement, and conversation/communication support. Table 3 gives an overview of the technologies studied with regard to their purpose, type, media contents and services, individualization process, environment of use, any training provided and the way in which the technology was facilitated in the study.

**Table 3.** Overview of technology, individualization, and facilitation.

Purpose	Type	Study	Name	Media contents and services	Individualization process	Setting	Training	Facilitation
Reminiscence or memory support	Lifelogging: wearable camera devices	De Leo et al. (2011)	–	Photos taken on smartphone lanyard	Photos taken of everyday life at 5-minute intervals. Photos then saved to DVD	Home	Given to people with dementia and caregiver by researchers	People with dementia uses smartphone independently, then watches DVD of photos with caregiver
		Karlsson et al. (2014, 2017)	SenseCam/digital photograph diary	Photos taken by wearable camera, captions with geolocation	Wearable camera (SenseCam) takes photos of daily events. Photos then uploaded onto touchscreen computer (Digital photograph diary)	Home	Given to people with dementia and family member by researchers	People with dementia uses device, family uploads photos. They review photos together
		Piasek et al. (2012)	SenseCam	Photos taken by wearable camera	Wearable camera takes photos of daily events. Photos then reviewed on computer	Home	Given to people with dementia and family by researchers Given to people with dementia by researchers	People with dementia uses device, images reviewed with therapist
		Silva et al. (2017)				Hospital/clinic		People with dementia uses device, images reviewed with neuropsychologist
		Woodberry et al. (2014)				–		People with dementia uses device, researcher or spouse uploads photos
	Digital app	Critten and Kucirkova (2017)	Our Story	Photos taken on iPad and found online, text captions, audio narration	Story captured in “conversational style” with people with dementia. People with dementia find own photos.	Home/club for people with dementia	Given to people with dementia by authors	Independent use by people with dementia (with researcher support if needed)
		Hashim et al. (2015)	myBook	Daily routine reminders, family photos, games	Caregivers supplied information and photos to authors	Home	Given to people with dementia and caregiver by authors	People with dementia and caregiver use together
		Laird et al. (2018)	InspireD	Photos, videos, music	People with dementia and family upload media contents with help of reminiscence trainer	Home	Given to people with dementia and family by IT assistant (+ reminiscence training)	People with dementia and family use together
		Ryan et al. (2018)			People with dementia and family upload media contents to app		–	
		Peeters et al. (2016)	Music ePartner	Music with text captions and photos	Questionnaire filled in by people with dementia/caregiver and given to authors	Day center	Given to people with dementia and caregiver by researcher	People with dementia and caregiver use together
	Multimedia biography (DVD/video-based)	Damianakis et al. (2010)	–	Photos, video, voice-over narration, music	Student collaboration with people with dementia and family using workbook	Home		People with dementia and family use together
		Park et al. (2017)	–	Photos, voice recordings, music, animation	People with dementia, Family and researcher	Community club	Given to people with dementia and family by authors	People with dementia and family use together guided by workshop facilitator
		Subramaniam and Woods (2016)	–	Photos, music, narration, video	Participatory design—people with dementia and Family, researcher as co-editor	Care home		Digital life book played on TV in people with dementia’s room
		Yasuda et al. (2009)	–	Photos, narration, music	Caregivers provided photos to researchers	Hospital		People with dementia watched by him/herself
	Touchscreen device	Massimi et al. (2008)	Biography theatre	Photos, music, video, narration	Authors worked with people with dementia and family	Home	–	Independent use by people with dementia
Stimulate engagement	Social robot	Khosla et al. (2014, 2016)	–	Music, photo, social connectivity, games	Real-time individualization by robot capturing emotional responses	Home	–	Use by people with dementia and family
	PowerPoint	Davis and Shenk (2015)	–	Photos, music, video	Students used photos provided by family	Memory care unit	–	Facilitated by student volunteer
	Digital app	McAllister et al. (2020)	Memory Keeper	Family photos, music, video, book covers, themed images	Researcher collaboration with people with dementia and family	Long-term care facility	Given to family by researchers	People with dementia and family member use together
Behavior management	Multimedia touchscreen device	Davison et al. (2016)	Memory Box	Movies, music, photos, video messages from family, family photos	Researcher collaboration with people with dementia and family	Nursing home	2.5 hours training given to people with dementia, staff, family by researchers	Main use by people with dementia, support from family and staff carers
		Kerssens et al. (2015)	The Companion	Photos, captions, music, reminders, messages	Life story interview with people with dementia and family	Home	Given to people with dementia and caregiver by expert in assistive technology	Use by people with dementia and family
		Navarro et al. (2015, 2016)	AnswerBoard/AnswerPad	Reminders, personalized games	Researcher collaboration with caregiver	Home	–	Use by people with dementia with therapist
	Tablet with video only	Hung et al. (2018)	–	Video from family	Researcher collaboration with family	Hospital	–	Used by professional carer
		O’Connor et al. (2011)	–	Video from family	Family pre-recorded messages	Care home	Given by researchers	Used by professional carer
Communication/conversation support	Digital app	Ekström et al. (2015)	GoTalk NOW	Photos, video, text captions, digitized and synthetic speech	Collaboration between people with dementia and speech therapist over 3 days	Home	Given to people with dementia and family member by researchers	Used by people with dementia and family
		Samuelsson and Ekström (2019)	CIRCUS	Photos, films, music, videos	Upload function on app	Home	Given by researchers to caregivers only	Used by people with dementia and professional caregiver
		Welsh et al. (2018)	Ticket to Talk	Photos, sounds, videos	Young person creates user profile with help from family. App provides prompts to invite young person to find out more about the people with dementia’s life	Home/care home	–	Used by young person, people with dementia and family/carer

Note: Symbol “–” indicates not specified.

##### Environment, training, and facilitation

The majority of studies (N = 18) were conducted within the homes of participants, who were living in the community (Critten & Kucirkova, 2017; Damianakis et al., 2010; De Leo et al., 2011; Ekström et al., 2015; Hashim et al., 2015; Karlsson et al., 2014, 2017; Kerssens et al., 2015; Khosla et al., 2014, 2016; Laird et al., 2018; Massimi et al., 2008; Navarro et al., 2015, 2016; Piasek et al., 2012; Ryan et al., 2018; Samuelsson & Ekström, 2019; Welsh et al., 2018). Family members were often the facilitator of the technology use. In most studies, the presence of another person was required for the full facilitation of the intervention/activity. Whether it be family member, professional caregiver or therapist, it was deemed important that the technology was used as a joint activity. Even in the case where the person with dementia was encouraged to use the device or app independently, support from caregivers was available. Therefore, training of the technology was often given to both the person with dementia and their caregiver. Most studies were quite vague about the instructions given. However, a few studies described extensive training procedures (Davison et al., 2016; Kerssens et al., 2015; Laird et al., 2018). For example, in the study of the InspireD app (Laird et al., 2018), an IT assistant provided training to participants with dementia and their family members, who were living at home. In addition to this, participants also received reminiscence training.

There were a couple of cases where the person with dementia was trained individually. Davison et al. (2016) reported that each of the 11 participants (with mild to severe dementia) received 2 hours of individual training to use a personalized multimedia touchscreen device. This training utilized spaced retrieval learning principles and involved research staff demonstrating procedures and asking the participant to imitate them. Despite this training, however, some participants were unable to use the device due to cognitive or sensory impairment. Similarly, participants (with mild to moderate dementia) in the study of the OurStory iPad app were trained to use the app independently, however they experienced practical difficulties such as not being able to hold the device or being unable to use the keyboard (Critten & Kucirkova, 2017).

Facilitation ranged from professional caregivers having complete control of the technology (e.g., simulated presence on iPad apps in Hung et al., 2018; O’Connor et al., 2011), to joint use between people with dementia and family members (e.g., multimedia apps in Laird et al., 2018; Ryan et al., 2018, digital life storybooks in Critten & Kucirkova, 2017; Park et al., 2017, social robots in Khosla et al., 2014, 2016), and to more independent use by the person with dementia (e.g., Biography Theatre in Massimi et al., 2008). The most independently used devices were the lifelogging technologies. The SenseCam (Karlsson et al., 2014, 2017; Piasek et al., 2012; Silva et al., 2017; Woodberry et al., 2014) and the smartphone lanyard used by De Leo et al. (2011) were worn by the person with dementia during the day. However, in all studies of lifelogging technologies, support was needed from another individual to upload the photographs onto a DVD or computer. Reviewing the photographs then became a joint activity.

#### How are these technologies individualized?

Most of the studies described the individualization process as a collaboration between the person with dementia, the family member, and often a researcher. While most studies were unclear on the length of time taken to individualize the technology, there were some that used several weeks for the process. For example, digital stories were created over a 6-week period in Park et al. (2017) and an average of 8.3 weeks in Subramaniam and Woods (2016).

Despite common collaboration between participants with dementia, family and researchers, approaches to individualizing the app/device still differed. Examples include structured workbooks (Damianakis et al., 2010), a chronological approach by listing major life chapters (Massimi et al., 2008), stories captured in a “conversational style” (Critten & Kucirkova, 2017), life story interview (Kerssens et al., 2015), questionnaire (Peeters et al., 2016), participatory design (Subramaniam & Woods, 2016), in-app prompts (Welsh et al., 2018), and participants uploading their own media content to apps (Laird et al., 2018; Ryan et al., 2018; Samuelsson & Ekström, 2019). The resonating theme among all these approaches is that of capturing the life story of the individual using photographs, music, and narratives (both textual and audio-recorded).

Several studies used theory to inform the individualization process. For instance, Positioning Theory (Harr & Van Langenhove, 1998) informed Karlsson et al.’s (2017) work on the SenseCam. The approach to the study was to understand narrations about recent events as being co-constructed between the person with dementia and their partner. Park et al. (2017) was influenced by Bruner’s paradigm of narrative knowing and constructivism (Bruner, 2003). Damianakis et al. (2010) was informed by a framework that supports coherence of ego integrity and personhood during phases of impairment. Finally, Ryan et al.'s (2018) work on the InspireD app was underpinned by Kitwood’s notion of person-centered care (Kitwood, 1997). These four studies that used theoretical foundations for narrative creation all reported positive effects on self-identity and/or engagement from qualitative methods including discourse analysis, interviews, observations and field notes.

#### What is known about the effects of these technologies on the well-being of people living with dementia?

Overall, the evidence from the included studies suggest that individualized, digital technologies can have positive effects on the well-being of people living with dementia. Particularly promising areas of improvement include behavior and mood, sense of identity, and relationships and engagement with others. Specific domains of well-being are reported on in further detail below.

##### Memory

The impact of these technologies on memory was mixed, and methods to assess memory were varied. Based on observational and interview data, several studies found that personalized multimedia can stimulate reminiscence (Critten & Kucirkova, 2017; Damianakis et al., 2010; Hashim et al., 2015; Welsh et al., 2018). The use of formal tests on memory was scarce. Two studies used the Autobiographical Memory Interview (AMI) (Kopelman et al., 1989), including Subramaniam and Woods (2016) who found that the use of a digital life storybook improved autobiographical memory after using the storybook for four weeks. Contrastingly, the study of a personalized biographical ambient display did not improve AMI scores after one month of use (Massimi et al., 2008). Mixed results were also present in the study of lifelogging technologies. De Leo et al. (2011) and Woodberry et al. (2014) found that pictures taken by a wearable camera enabled the participants to recall significantly more details of recent events, as measured by non-standardized memory recall tests. However, a single case study of SenseCam conducted by Piasek et al. (2012) reported that the participant was confused about the source of images. Karlsson et al. (2017) also reported that there were certain situations where participants with Alzheimer’s disease were unable to recall any information related to the event shown from SenseCam photographs.

##### Behavior and mood

Overall, the technologies included for review showed beneficial effects on behavior and mood. Furthermore, studies that focused on this domain were more consistent in using standardized outcome measures. The AnswerBoard (public ambient display) and AnswerPad (mobile phone app) devices were shown to have a positive effect in reducing challenging behaviors after 16 weeks of use, as indicated by decreased NPI-Q scores (Navarro et al., 2015, 2016). Another study on an ambient display system used the Apathy Evaluation Scale and found that the use of the system reduced the participant’s apathy after one month of use (Massimi et al., 2008). A personalized multimedia system was shown to significantly reduce depression and anxiety after four weeks of use, as measured by the CSDD and RAID (Davison et al., 2016). Silva et al. (2017) found that the use of the SenseCam significantly reduced depression scores, using the Geriatric Depression Scale.

Studies focusing on simulated presence to reduce problematic behaviors had positive results. O’Connor et al. (2011) found that presenting residents with an iPad containing a video-recorded message from a family member for 14 days was able to significantly reduce resistance to care. Similarly, Hung et al. (2018) also tested iPad-facilitated video simulated presence for 14 days and found that hospital patients with dementia responded positively. As well as improving behavior, the iPad intervention also resulted in positive changes in the mood of all participants. However, the authors noted that video content with too many family members with multiple messages provoked anxiety, emphasizing the importance of acknowledging the individual needs of the person with dementia and being aware of possible over stimulation.

##### Self-identity

Results from the identified studies suggest that a sense of self can be preserved, even in later stages of dementia. Critten and Kucirkova (2017) found that the Our Story app gave participants confidence, empowerment and increased self-esteem. Karlsson et al. (2017) studied the SenseCam in relation to self and identity. From discourse analysis, the authors identified two key themes: manifestations of sense of self and self in relation to others. With regard to sense of self, the authors found that even if the participant could not relate to the event shown in the photograph, the material still stimulated conversation about personal experience. When the participants’ partners had been involved in events captured by the SenseCam, narrating and remembering the event became a joint activity. However, it is important to note that some participants became stressed when the conversation became interrogative. The only study to use outcome measures for self-identity was Massimi et al. (2008) who used the Twenty Statements Test and the Self Image Profile (Adult). The authors found that use of the Biography Theatre for one month led to an improvement in positive self-identity. It is also important to note that studies which included the person with dementia in the individualization process of the technology empowered the individual to become more connected with their sense of self. For example, participants in a digital storytelling workshop enjoyed the process of creating and sharing their stories over a six-week period (Park et al., 2017).

##### Social relationships and engagement

Given the highly interactive nature of the technologies, many studies found improvements in relationships, communication and engagement. Social robots were identified as a way of facilitating engagement and interaction for people with dementia (Khosla et al., 2014, 2016). Personalized digital media was considered as a tool for starting conversations (Davis & Shenk, 2015; Karlsson et al., 2014; Samuelsson & Ekström, 2019; Yasuda et al., 2009), supporting interaction (Hashim et al., 2015; Park et al., 2017), and improving relationships between people with dementia and their caregivers (Karlsson et al., 2014; Laird et al., 2018; Park et al., 2017; Ryan et al., 2018). It was also reported that such media provided caregivers, and sometimes even family members, with new insights and heightened perspectives into the life of the person with dementia (Damianakis, 2010; Ryan et al., 2018; Samuelsson & Ekström, 2019). The majority of these findings were based on interview or observation data. However, Laird et al. (2018) used the Quality of Carer and Patient Relationship scale (Spruytte et al., 2002) and the Mutuality Scale (Archbold et al., 1990) to assess the effect of the InspireD app on the relationship between the person with dementia and their caregiver. Scores on both scales were significantly improved after 12 weeks of using the iPad app.

There were some cases where tensions in the relationship were reported. For example, Ekström et al. (2015) found that problems associated with dementia were foregrounded during joint interaction with a tablet computer. The person with dementia became dependent on their conversational partner to be able to use the technology. Similar issues were experienced with the SenseCam. The participant in Piasek et al.’s (2012) study of SenseCam relied on his wife while reviewing photographs together with a therapist. Participants in another SenseCam study were reportedly frustrated when they felt the conversation about the photographs had become a test of their memory (Karlsson et al., 2017).

##### Emotional well-being

Observations of interaction with technology were used to assess emotional reactions from the participants. Technologies that featured reminiscence activities or other autobiographical material provided participants with an enjoyable experience (Critten & Kucirkova; Damianakis et al., 2010; Hashim et al., 2015; Kerssens et al., 2015; Khosla et al., 2014, 2016; McAllister et al., 2020; Park et al., 2017; Peeters et al., 2016; Ryan et al., 2018; Samuelsson & Ekström, 2019; Subramaniam & Woods, 2016). However, due to the highly personal nature of these activities, there is a potential for sensitive topics to cause negative reactions. There were numerous reports of sadness being experienced, especially when personal photographs of those who had passed away were used (Damianakis, 2010; Ryan et al., 2018). In these cases, it is important to remember that emotions are highly complex. Damianakis et al. (2010) commented on the possibility of observing both happiness and sadness simultaneously in reaction to pictures of a deceased loved one. Furthermore, family members involved in the study felt that it was important to include photographs and stories of loved ones, even if they had passed away.

## Discussion

### Summary of evidence

This review has identified the varying types of digital technologies that are being used to create individualized, meaningful activities for people with dementia. Overall, the findings suggest the use of individualized technology to be promising in contributing to and advancing dementia care. Technology can be used to complement psychosocial approaches to care such as reminiscence therapy, simulated presence therapy, occupational therapy and life story work. Additionally, this review has demonstrated how theory-based knowledge may be used to complement technology-based activities in dementia care. Studies that used theoretical foundations for the individualization process of the technology all found positive impacts on a sense of self and/or engagement, suggesting that theory-based knowledge can be beneficial for technology development.

Findings from the review also indicate the amount of progress that has taken place in this field. Only 7 of the 29 included studies did not actively involve the person with dementia in the individualization process of the technology. This contrasts to a 2008 literature review on technology studies to meet the needs of people with dementia and their caregivers. Topo (2008) found that very few studies actively involved the person with dementia in using the technology. Studies identified in this review not only involved the person with dementia as users of the technology, but in most cases, they were involved in the individualization process, acting as co-creators of their own narratives. There was also a case where the individuals with dementia were involved in the development of the technology itself. The InspireD app was co-created by a User Development Group that consisted of six people with dementia working together with researchers (Laird et al., 2018; Ryan et al., 2018). Future work in this area should adopt a similar approach, involving people with dementia as co-creators from the onset of the technology development.

#### Opportunities afforded by technology

Findings from this review are in accordance with other literature reviews in this area, in terms of the benefits that technology can provide to people living with dementia. A systematic review of technology for reminiscence therapy found that using information and communication technologies for reminiscence therapy interventions has benefits such as providing access to rich multimedia materials, providing opportunities for social interaction, provision of memory support and ownership of conversations (Lazar et al., 2014). Similar results are resonated in this review, especially with regard to social interaction. Furthermore, the use of technology to preserve, share and explore the narrative of the person with dementia is consistent with earlier findings in this area (Maiden et al., 2013; Purves et al., 2011).

One particularly meaningful benefit of technology is that it provides a means of being able to access a wealth of images and other types of media. This can be very important, especially for those who may not have many photographs from their past. Participants using the OurStory iPad app found access to external images important (Critten & Kucirkova, 2017). This continuous and endless access to media also provides an opportunity to engage with not just the past, but also the present. Participants using the InspireD app were able to take pictures on the iPad and include them as part of their reminiscence program (Laird et al., 2018; Ryan et al., 2018). SenseCam captures everyday moments of daily life, enabling people to recollect upon recent events. The upload feature in CIRCUS (Samuelsson & Ekström, 2019) also allows participants to engage with media from recent events, if they wish to do so.

Technology also presents life histories in a new way, which can be beneficial for all individuals involved its use. Participants in the study of a digital life book were excited about seeing their life history: “I feel like I’m famous. I feel very excited,” “I can’t believe this, my mother will be proud of me . . I feel like I’m being appreciated” (Subramaniam & Woods, 2016). Additionally, caregivers felt that technology provided a way of learning more about the life of the person with dementia (Damianakis et al., 2010; Ryan et al., 2018; Samuelsson & Ekström, 2019; Subramaniam & Woods, 2016). As Purves et al. (2011) suggest, technology can be a way of shaping an interactional environment in which narrative can be explored together: “With these technologies at our disposal, we not only have better ways to elicit and convey narratives … but we also have better ways to share these narratives with others, over time and across place” (p. 240). The technologies identified in this review provide examples of how this may be achieved.

#### Challenges going forward

The results from this review have raised some potential issues that could be faced when implementing individualized technologies into practice. Associated costs are an important issue. The Memory Box device (Davison et al., 2016) cost 12,000 U.S. dollars for four units. Installation of the Biography Theatre took an experienced technologist 30–40 hours over the course of one month (Massimi et al., 2008). In Laird et al.’s (2018) study of the InspireD app, which itself is free, the training sessions cost 2750 GBP per dyad.

Most studies were conducted in the homes of people with dementia, and this may be due to the fact that support from another individual was often needed in order to be able to use the technology. Care institutions such as nursing homes are often busy environments, in which one-to-one interaction may not always be possible due to time constraints. Additionally, home-dwelling individuals with dementia tend to be in more mild–moderate stages of dementia and therefore may be able to use the technology on a more independent level. This then raises the question of how practical it is to introduce such technology into care homes for individuals in more severe stages of dementia. Also, the issue of capacity was raised in Subramaniam and Wood’s (2016) study of the digital life book. It was questioned whether members of staff could be expected to take on the role of coproducing the life stories together with the person with dementia.

Additionally, and maybe most importantly, there is a question of how well these technologies can be introduced to this vulnerable group, especially in later stages of dementia. Numerous participants across the studies experienced difficulties in being able to interact with the technology. Examples include physical issues with being able to hold the device or press buttons, issues with being able to see the screen, or difficulties with general operation of the technology. Piasek et al. (2012) witnessed a particular struggle with SenseCam in getting the participant, John, to remember that he was the one wearing the camera: “The SenseCam technology seemed confusing for someone with such severe memory impairments. It also seemed pointless to continuously explain what SenseCam is and that is was John who wore it.” Even in studies where participants were aware of the SenseCam, they did not always respond positively to it. For example, one gentleman felt embarrassed by wearing the camera and felt it drew attention to him (Woodberry et al., 2014). These issues highlight the need to continue to develop awareness in potentially problematic areas such as physical limitations or sensory issues as well as self-consciousness or stigma. It is important that devices and technologies are developed with these issues in mind, so that they may be feasible for use by the target population.

### Limitations

This is a relatively new field of research and new technologies are constantly being presented, meaning that the evidence on its impact and effectiveness is still somewhat limited. The findings from this review are limited by the small sample sizes of the included studies. Given the amount of time and effort required for individualizing technologies, especially when the identification and collection of personalized multimedia is involved, it is understandable that most studies had small sample sizes. Seven studies included only one participant. While these small case studies are valuable for providing rich, in-depth accounts, the findings are hard to generalize to a wider population. There is a clear need for studies with larger sample sizes with standardized outcome measures. Additionally, the time of use of the devices was highly varied among the studies. The use of the technology ranged from single-time use to six months.

Another limitation of this review is the lack of a quality appraisal of the included studies. Given the fact that the use of individualized technologies in dementia care is still an emerging field, we wanted to include a variety of studies in order to gain a broad overview of the topic. Most of the research in this area consists of small case studies, and excluding these studies based on their quality would have resulted in a limited understanding of how these technologies can be potentially used in dementia care. There is some level of quality of assurance, given the fact that only articles from peer-reviewed journals were included for review. However, there may be potential bias from studies where researchers acted as collaborators or co-editors in the individualization process. For example, participants in Critten and Kucirkova’s (2017) study of the Our Story app enjoyed the process of reminiscing together with the first author and commented that the activity had brought back some ‘happy times’ that the participants were keen to share with the researcher at later interviews. Massimi et al. (2008) stated that a relationship had developed between the participant and the researcher in their role as “biographer.” The authors state that this relationship shifted focus from the participant being “an old man with a bad memory” to being a human being. However, this is to be expected, given that participatory design and co-creative approaches are increasingly being adopted in dementia research. Once more knowledge exists in this area, there will be a need to critically evaluate the quality of the evidence.

Finally, a considerable number of articles had to be excluded for review due to lacking reports on the effects on the well-being of the person with dementia. This meant that other emerging technologies in this area were not commented on. It is important to be aware of other technologies beyond those included in this review, and how they can also create opportunities for the conveying of narrative. For example, virtual reality can be a means of recreating environments from the past (Hodge et al., 2018). Another example is a project called SENSE-GARDEN, which is developing multisensory spaces that combine music, film, pictures, and scent with innovative technology to create an immersive environment tailored specifically for the individual with dementia (Goodall et al., 2019).

## Conclusion

Various technologies can add value to the individualization of meaningful activities in dementia care. This review highlights the need to focus on how these types of technologies could potentially be implemented into care practice, particularly in nursing home environments. Previous reviews of technology studies have raised issues that are still present today, with this review showing that studies are still highly varied in terms of design, sample sizes, methods of assessment, and the type of technology being used.

This review has also highlighted several important aspects to bear in mind when developing technologies for people with dementia. Findings suggest that people with dementia are able to learn how to use new technologies in more severe stages of dementia; however, support from caregivers is likely to still be needed. In order to further inform practice, future studies should assess time consumption, training requirements, costs, and long-term benefits. It is also important that technology is used as means to support people with dementia in fulfilling meaningful occupation, rather than as a means of interrogation. By developing technology in a user-friendly and user-conscious way, ideally with direct involvement of people with dementia, the right balance between support and empowerment can be identified.

To conclude, this review suggests that the use of individualized, digital technologies can have a positive impact on the well-being of people living with dementia. The included studies provide valuable information on how to individualize and facilitate the use of such technologies, which may serve as useful recommendations for implementing these technologies into practice and conducting future research. However, given the methodological limitations of research conducted in this area, more work is needed to strengthen the evidence base for using individualized, digital technologies in dementia care.

## Supplemental Material

sj-pdf-1-dem-10.1177_1471301220928168 - Supplemental material for The use of technology in creating individualized, meaningful activities for people living with dementia: A systematic review Click here for additional data file. Supplemental material, sj-pdf-1-dem-10.1177_1471301220928168 for The use of technology in creating individualized, meaningful activities for people living with dementia: A systematic review by Gemma Goodall, Kristin Taraldsen and J Artur Serrano in Dementia
