# Supplementary material for: The use of technology in creating individualized, meaningful activities for people living with dementia: A systematic review
Source: Dementia (London). 2020 May 31;20(4):1442–69. doi: 10.1177/1471301220928168 (PMC8132010; doi:10.1177/1471301220928168)
Supplement: sj-pdf-1-dem-10.1177_1471301220928168 - Supplemental material for The use of technology in creating individualized, meaningful activities for people living with dementia: A systematic review [file sj-pdf-1-dem-10.1177_1471301220928168.pdf]

Supplementary material

Table S1. Overview of outcome measures used in the included studies

Domain	Measure	Studies	Total
BPSD	Geriatric Depression Scale (GDS)	Massimi et al. (2008); Silva et al. (2017); Subramaniam and Woods (2016)	3
	Apathy Evaluation Scale (AES)	Massimi et al. (2008); Navarro et al. (2015; 2016)	3
	Neuropsychiatric Inventory (NPI)	Navarro et al. (2015; 2016)	2
	Cohen-Mansfield Agitation Inventory (CMAI)	Davison et al. (2016)	1
	Cornell Scale for Depression in Dementia (CSDD)	Davison et al. (2016)	1
	Rating Anxiety in Dementia (RAID)	Davison et al. (2016)	1
	Observational Measurement of Engagement	Davis and Shenk (2015)	1
	Positive response schedule	O'Connor et al. (2011)	1
Memory	Autobiographical Memory Interview (AMI)	Massimi et al. (2008); Subramaniam and Woods (2016)	2
	Free recall memory index	Karlsson et al. (2014)	1
	Recognition memory index	Karlsson et al. (2014)	1
Cognition	Mini-mental state examination (MMSE)	Karlsson et al. (2014); Massimi et al. (2008)	2
	Informant Questionnaire of Cognitive Decline in the Elderly (IQCODE)	Massimi et al. (2008)	1
Well-being	Philadelphia Geriatric Centre Morale Scale (PGCMS)	Karlsson et al. (2014)	1
	WHO-5 Well-being Index	Laird et al. (2018)	1
Relationships	Mutuality Scale	Laird et al. (2018)	1
	Quality of the Carer Patient Relationship (QCPR)	Laird et al. (2018)	1
Quality of Life	WHO Quality of Life- Old (WHOQOL-OLD)	Silva et al. (2017)	1
	Quality of Life in Alzheimer's Disease (QOL-AD)	Subramaniam and Woods (2016)	1
Identity	Self-image Profile Adult (SIP-AD)	Massimi et al. (2008)	1
	Twenty Statements Test (TST)	Massimi et al. (2008)	1
Goal setting	Goal Attainment Scale (GAS)	Massimi et al. (2008)	1
Functioning	Adults and Older Adults Functional Assessment Inventory (IAFAI)	Silva et al. (2017)	1
